# Can sedentary behavior be made more active? A randomized pilot study of TV commercial stepping versus walking

**DOI:** 10.1186/1479-5868-9-95

**Published:** 2012-08-06

**Authors:** Jeremy A Steeves, David R Bassett, Eugene C Fitzhugh, Hollie A Raynor, Dixie L Thompson

**Affiliations:** 1 Cancer Prevention Fellowship Program, National Cancer Institute, 6120 Executive Boulevard, Bethesda, MD, 20892, USA; 2 Department of Kinesiology, Recreation, and Sport Studies, University of Tennessee, Knoxville, TN, USA; 3 Department of Kinesiology, Recreation, and Sport Studies, University of Tennessee, Knoxville, TN, USA; 4 Department of Nutrition, University of Tennessee, Knoxville, TN, USA; 5 Department of Kinesiology, Recreation, and Sport Studies, University of Tennessee, Knoxville, TN, USA

**Keywords:** Walking, Physical activity intervention, Obesity, Diet, Weight, Behavior change, TV commercial stepping

## Abstract

**Background:**

There is a growing problem of physical inactivity in America, and approximately a quarter of the population report being completely sedentary during their leisure time. In the U.S., TV viewing is the most common leisure-time activity. Stepping in place during TV commercials (TV Commercial Stepping) could increase physical activity. The purpose of this study was to examine the feasibility of incorporating physical activity (PA) into a traditionally sedentary activity, by comparing TV Commercial Stepping during 90 min/d of TV programming to traditional exercise (Walking).

**Methods:**

A randomized controlled pilot study of the impact of 6 months of TV Commercial Stepping versus Walking 30 min/day in adults was conducted. 58 sedentary, overweight (body mass index 33.5 ± 4.8 kg/m^2^) adults (age 52.0 ± 8.6 y) were randomly assigned to one of two 6-mo behavioral PA programs: 1) TV Commercial Stepping; or 2) Walking 30 min/day. To help facilitate behavior changes participants received 6 monthly phone calls, attended monthly meetings for the first 3 months, and received monthly newsletters for the last 3 months. Using intent-to-treat analysis, changes in daily steps, TV viewing, diet, body weight, waist and hip circumference, and percent fat were compared at baseline, 3, and 6 mo. Data were collected in 2010–2011, and analyzed in 2011.

**Results:**

Of the 58 subjects, 47 (81%) were retained for follow-up at the completion of the 6-mo program. From baseline to 6-mo, both groups significantly increased their daily steps [4611 ± 1553 steps/d vs. 7605 ± 2471 steps/d (TV Commercial Stepping); 4909 ± 1335 steps/d vs. 7865 ± 1939 steps/d (Walking); P < 0.05] with no significant difference between groups. TV viewing and dietary intake decreased significantly in both groups. Body weight did not change, but both groups had significant decreases in percent body fat (3-mo to 6-mo), and waist and hip circumference (baseline to 6-mo) over time.

**Conclusions:**

Participants in both the TV Commercial Stepping and Walking groups had favorable changes in daily steps, TV viewing, diet, and anthropometrics. PA can be performed while viewing TV commercials and this may be a feasible alternative to traditional approaches for increasing daily steps in overweight and obese adults.

**Trial Registration:**

This study is registered at ClinicalTrials.gov, NCT01342471

## Background

According to the 2008 Physical Activity (PA) Guidelines for Americans, to receive the health benefits of exercise, adults should accumulate at least 150 min/wk of moderate-intensity PA, ideally spread out over several days of the week and accumulated in bouts of 10 min or longer [1]. Unfortunately, more than half of all adults do not exercise enough to meet these guidelines, and approximately 25% of the population report being completely sedentary during their leisure time [2].

For many inactive individuals, increasing PA can be a challenge, and initiating PA can be intimidating [3-5]. Gaining support from others and finding activities that are enjoyable are critically important to help a person progress towards the recommended levels of PA [3]. However, “lack of time” is one of the most frequently reported barriers preventing individuals from meeting PA guidelines [6,7]. In contrast, American adults spend an average of 3–5 h/d (up to 35 h/wk) viewing television [8-10]. TV viewing is the most common leisure-time sedentary activity in America [10].

Developing strategies that assist inactive individuals in engaging and sustaining regular activity patterns is a priority for health professionals because of the health risk reduction associated with the transition from being inactive to attaining the recommended amount of PA [3-5,11]. Because of the nature of the dose–response relationship between PA and disease-related conditions, the greatest public health benefits are gained as people move from being sedentary to reaching minimal PA recommendations [12,13]. Independent of PA, decreasing sedentary behaviors is an important public health focus gaining attention [14-16]. Both increased participation in PA and a reduction of sedentary behaviors have an important role in promoting a healthy lifestyle [17,18]. For inactive adults, stepping in place during television commercials (TV Commercial Stepping) may be an effective method to increase moderate-intensity PA and simultaneously combat the negative effects of sedentary TV viewing.

Previously, a laboratory-based study established that stepping in place is a moderate-intensity activity [19], and that stepping in place can be accurately recorded with pedometers [20]. During 60 min of watching TV with TV Commercial Stepping, participants accumulated approximately 2100 steps on commercial breaks [19]. The standard half-hour TV program contains 8–12 minutes of commercials, thus TV Commercial Stepping during 90 min of TV programming (~24-36 min of commercials) should approximate nearly the same volume of PA as 30 min of continuous walking. Walking at a moderate pace (2.8 to 3.2 mph) is also a moderate intensity activity (3.5 METS) [21].

To date, we are unaware of any randomized trials that have examined the effectiveness of TV Commercial Stepping to increase PA. Thus, the aim of this pilot study was to examine the feasibility and short-term efficacy of a novel intervention to increase PA over a 6-mo period. Subjects were randomized to: (1) TV Commercial Stepping during 90 min/d of TV programming, or (2) a traditional PA recommendation of walking at least 30 min/d. Outcomes included objectively measured PA (measured as steps/d), self-reported PA participation, TV viewing time, dietary intake, and anthropometrics. Feedback about the feasibility of the intervention among participants was also assessed.

## Methods

### Participants

Overweight and obese adults, recruited by flyers and newspaper advertisements, were enrolled in a university-based PA program during the months of September through November, 2010. Eligibility criteria included being between 35 and 65 years of age, having a BMI between 25 and 45 kg/m^2^, viewing ≥14 h per wk of TV, and having the ability to walk 1/4 mile without stopping. Participants were excluded if they reported physical or medical limitations for engaging in PA; had a resting blood pressure greater than 180 mm Hg systolic and/or 100 mm Hg diastolic; did not have a television in the home; or were currently participating in a program to increase PA. There was no racial or gender bias in the selection of participants. Participants signed the written informed consent and were randomized into either the TV Commercial Stepping group or the Walking group. Individual random assignment was determined by a computer based random number sequence. The allocation ratio was 1:1, with a fixed block size of 4, and was not stratified. The study was approved by the Institutional Review Board at The University of Tennessee, Knoxville, TN and was registered at ClinicalTrials.gov (NCT01342471). Participants were compensated $50.00 for completing the study.

The novelty of the TV Commercial Stepping approach as a PA prescription limits our ability to perform a traditional power analysis. In a previously published study done in our laboratory, Hultquist et al., [22] was able to find a significant difference (P < 0.001) between daily steps when comparing 58 sedentary women randomly assigned two similar PA recommendations (10 K steps a day, or 30-minutes of walking a day). The 10 K a day group adhered to their prescription more and had significantly greater steps than the 30-minute group. A recent systematic review of 8 randomized controlled trials of pedometer-based physical activity interventions showed that physical activity levels increased by 2183 steps per day over baseline (95% CI, 1571–2796, P < 0.001) with sample sizes ranging from 16 to 58 total participants [23]. Using these sample size results as guidance, a recruitment goal of 60 participants was set.

### Study design

In this 6-month pilot study, both treatment groups were prescribed a similar volume of home-based exercise (≥ 150 min/wk). The two groups differed in the way that PA was prescribed.

*Walking group*. Twenty-nine participants were instructed to walk “briskly” for at least 30 min at least 5 d/wk. Participants built up to walking 30 min/d over the first 3 weeks; increasing duration from 10 min/d in week 1, to 20 min/d in week 2, to 30 min/d for the remainder of this study. Participants were instructed to walk for 30 min continuously or break their walking up into bouts of at least 10 min.

*TV Commercial Stepping group*. Twenty-nine participants were instructed to stand and “briskly” step in place, or “briskly” walk continuously around the room/house for the duration of each commercial break during at least 90 min of TV programming at least 5 d/wk. Participants were instructed to step in place at a “moderate pace” (e.g., 100–120 steps per minute), with each foot stepping up off the ground about 15–20 cm [19]. Participants reviewed appropriate stepping-in-place or walking around the room pace and technique during each the first 3 face-to-face meetings. Participants performed multiple (~9-12), short (~3-5 min) bouts, conveniently incorporated into their daily TV viewing time. Participants increased their TV Commercial Stepping by incorporating it into 30 min of TV programming per day during week 1, 60 min per day during week 2, and 90 min per day during the remainder of the study.

### Common treatment components

During the first 3 months, all participants attended monthly individual treatment meetings (60 min), and received monthly individual phone call sessions (30 min) led by a single investigator (JAS). During months 3 through 6, the monthly phone call treatment sessions continued, and individual meetings were replaced with monthly newsletters. These interactions focused on behavioral strategies (goal setting, self-monitoring, stimulus control, pre-planning, problem solving, cognitive restructuring, and relapse prevention) for modifying exercise behaviors. No specific advice about changing diet was given, and participants were asked to hold other lifestyle factors (e.g., diet and other PA) constant.

### Assessment procedures

Assessments were completed at baseline, 3, and 6 months. Participants recorded their daily steps measured using the Omron HJ 303 tri-axial accelerometer-based pedometer (Omron Healthcare, Inc. Bannockburn, Illinois) worn on the lower leg just above the ankle. This pedometer and placement was previously validated for use while stepping in place [20]. All participants, regardless of group assignment, wore the Omron pedometer for the duration of the 6-mo program. Subjects also reported daily PA in an activity log that was collected by the investigators at each scheduled visit. These logs were also used to record time spent viewing TV.

Dietary intake was assessed using a 3-d food record (2 weekdays and 1 weekend day). Participants were asked to record all food and drink consumed throughout the day, and indicate any consumption that occurred specifically while viewing TV (TV-related energy intake, and TV-related percent energy from fat). Dietary data were analyzed using Nutrition Data System Software for Research (NDSR) developed by the Nutrition Coordinating Center, University of Minnesota, Minneapolis.

Height was measured (without shoes) with a wall-mounted stadiometer. Weight and body composition were measured using a computer-based electronic scale (Tanita BC-418 body composition analyzer) that gives readings to the nearest 0.1 kg. The scale was calibrated using known weights, prior to data collection each day. Waist and hip circumferences were measured using a Gulick tape measure fitted with a tension handle [24]. Three measurements were taken at each site, and the average of the measurements was used for data analysis.

At the end of the 6-mo PA program, participants were asked to complete a short questionnaire regarding their reaction to the intervention, the acceptability of the PA program, their willingness to continue using the PA strategies, and any problems/issues that arose during the intervention.

### Statistical analysis

SPSS version 17.0.0 for Windows (SPSS Inc., Chicago, Illinois) was used for statistical analysis. Descriptive statistics were calculated for all baseline measures. Baseline differences between the groups were examined using independent t-tests or *X*^2^ analysis. Models were structured such that treatment comparisons were made using the intention-to-treat principle. A standard method that is contained in SPSS was used for imputing missing data. Specifically, missing values were imputed by generating five random values from a normal distribution that has a mean equal to the baseline value and variance equal to the estimated variance for the value of other participants at the time where the value is missing. Effects were computed by averaging the appropriate regression coefficient across models. A mixed-factor ANOVA (with time point as the within-participant variable, and group as the between-participant variable) was used to analyze changes in steps per day, PA participation, TV viewing time, overall energy and dietary fat intake, energy and dietary fat intake while viewing TV, and anthropometric variables. For significant interactions, pair-wise comparisons, using Bonferroni corrections, were conducted at each time point to determine when group differences occurred. All data are presented as means ± SD unless otherwise stated. For all statistical analyses, an alpha level of 0.05 was used to show significant differences.

## Results

**Figure 1:**
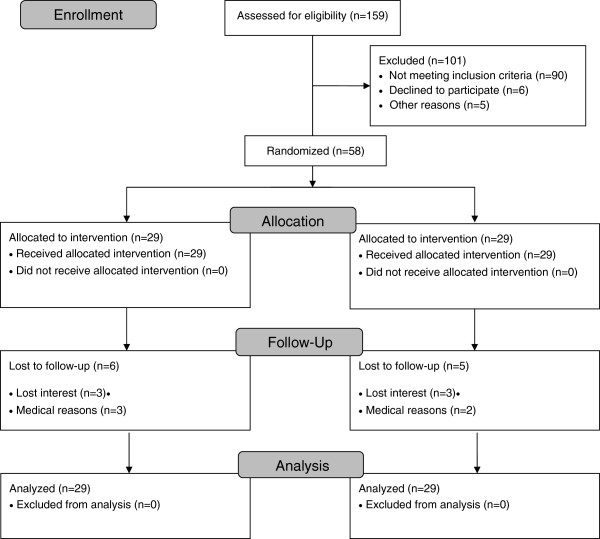
Participant Flow

A total of 58 individuals met eligibility criteria and agreed to participate in the study (Table 1). Participant flow through the study is presented in a CONSORT flow chart (Figure 1). Of the 58 subjects who participated in the study, 56 were white, and 2 were African American. Compared to the participants who were retained at 6-mo, the participants (n = 11) who did not return for the 6-mo assessment visit were significantly younger (45.4 ± 11.5 y), had greater BMI (36.6 ± 5.0 kg/m^2^), greater hip circumference (128.0 ± 13.4 cm), greater percent body fat (46.4 ± 6.5%), reported less walking (0.83 ± 1.5 min/d), and had lower average daily steps (3095 ± 964 steps/d) (P < 0.05).

**Table 1:** Baseline demographic and leisure-time characteristics of participants, mean ± SD

	**All participants (n = 58)**	**TV commercial step (n = 29)**	**30-min walk (n = 29)**
Sex	12 M/46 F	5 M/24 F	7 M/22 F
Age (years)	52.0 ± 8.6	53.8 ± 6.8	50.2 ± 9.8
Height (cm)	167.1 ± 7.9	166.0 ± 8.0	168.2 ± 7.8
Weight (kg)	93.8 ± 16.1	94.3 ± 17.5	93.3 ± 14.9
BMI (kg/m^2^)	33.5 ± 4.8	34.2 ± 5.5	32.8 ± 3.9
Percent fat (%)	41.8 ± 6.6	42.4 ± 6.9	41.3 ± 6.3
Waist circum. (cm)	103.8 ± 11.2	105.4 ± 13.6	102.3 ± 8.1
Hip circum. (cm)	120.3 ± 11.1	121.6 ± 12.3	118.9 ± 9.7
PA (steps/day Omron)	4760 ± 1443	4611 ± 1553	4909 ± 1335
TV viewing (hr/day)	4.2 ± 1.8	4.2 ± 1.5	4.1 ± 2.0

Circum. = circumference, F = female, M = male, PA = physical activity. There were no significant group differences.

**Figure 2:**
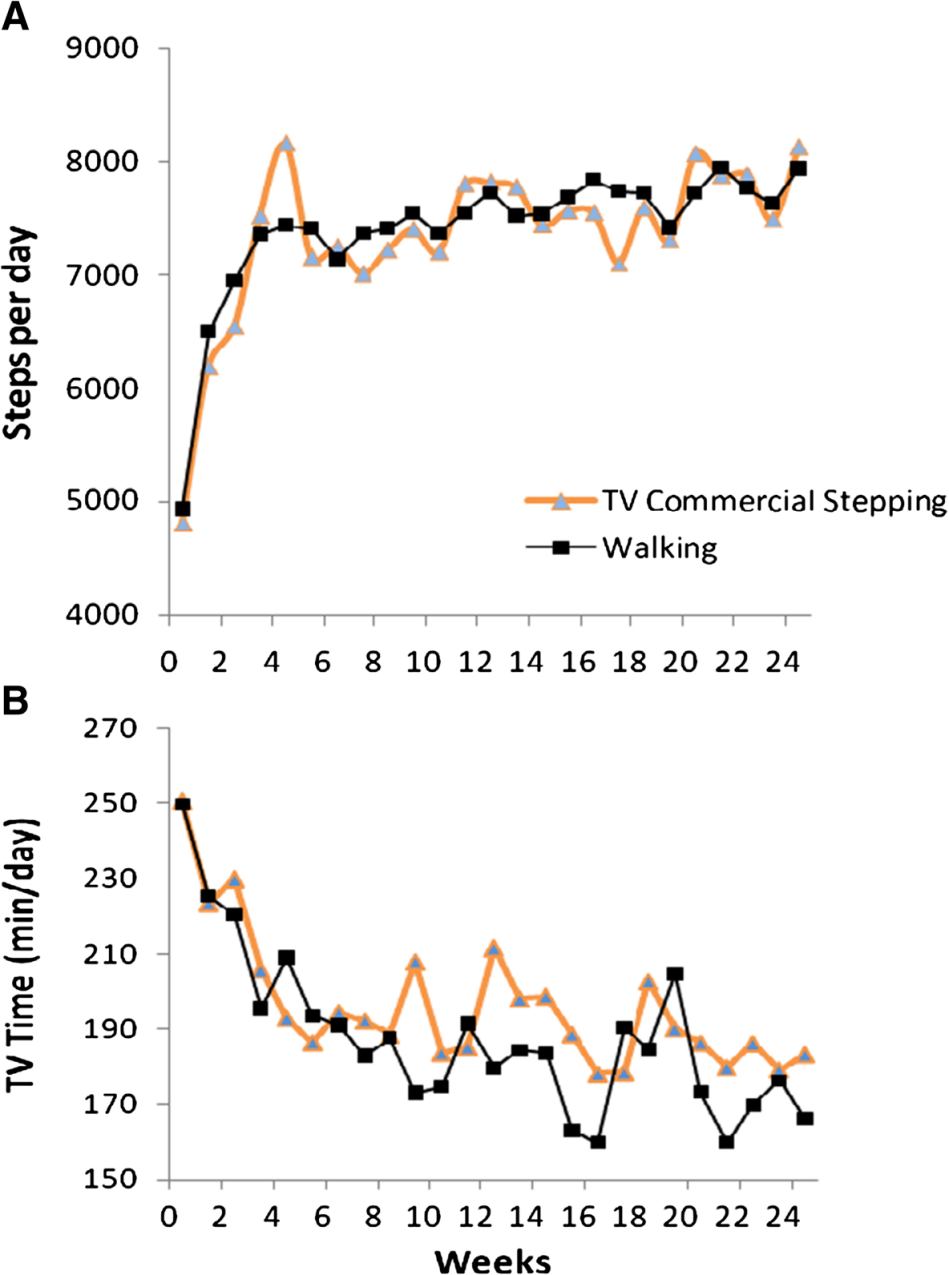
**Changes in average Omron-determined steps per day (A) and self-reported TV viewing (B) according to intervention group.** Note: Each data point represents the mean values for all participants examined at that time

Changes in average Omron-determined steps per day by group for each week of the intervention are shown in Figure 2A. Participants in the TV Commercial Stepping and Walking groups increased their PA by about 3000 steps/day from baseline (P < 0.05), with no differences between groups (Table 2). At the 3-mo and 6-mo assessment, participants in both groups met their PA goals more than 4 d/wk, and there was no difference between groups (Table 2). Self-reported TV viewing significantly decreased over time in both groups (P < 0.05) (Figure 2B). By 6 mo, participants in both groups watched about 1 hour less TV per day compared to baseline (Table 2).

**Table 2:** Physical activity and anthropometric characteristics of TV Commercial Stepping (n = 29) and Walking (n = 29) participants, data are mean ± SD

**Variable**	**Group**	**Baseline**	**3 mo**	**6 mo**
Total Steps (steps/day)	TV Stepping Walking	4611 ± 1553 4909 ± 1335	8078 ± 2306* 7562 ± 1901*	7605 ± 2471* 7865 ± 1939*
PA goals met (days/week)	TV Stepping Walking	0.0 ± 0.0 0.0 ± 0.0	4.2 ± 1.9* 4.3 ± 2.1*	4.3 ± 2.0* 4.4 ± 2.1*
TV Time (hr/day)	TV Stepping Walking	4.2 ± 1.5 4.1 ± 2.0	3.5 ± 2.1*^a^ 3.1 ± 1.7*^a^	3.0 ± 1.5* 2.7 ± 1.4*
Total energy intake (kcals/day)	TV Stepping Walking	1971.7 ± 642.5 2071.6 ± 528.7	1700.9 ± 500.3* 1725.8 ± 459.1*	1623.8 ± 386.5* 1717.4 ± 465.2*
TV-related energy intake (kcals/day)	TV Stepping Walking	643.3 ± 399.7 808.8 ± 455.1	367.5 ± 301.6* 459.9 ± 492.8*	360.7 ± 330.0* 292.2 ± 278.7*
Total percent energy from fat (%)	TV Stepping Walking	37.7 ± 6.9 35.7 ± 6.8	34.1 ± 6.7 36.7 ± 8.7	34.9 ± 7.0 36.7 ± 6.4
TV-related percent energy from fat (%)	TV Stepping Walking	36.9 ± 13.8 39.3 ± 13.5	23.5 ± 17.6* 31.9 ± 18.1*	27.0 ± 18.0* 30.3 ± 17.9*
Weight (kg)	TV Stepping Walking	94.3 ± 17.5 93.3 ± 14.9	94.6 ± 17.8 93.6 ± 15.4	93.6 ± 17.7 92.9 ± 14.9
BMI (kg·m^-2^)	TV Stepping Walking	34.2 ± 5.5 32.8 ± 3.9	34.3 ± 6.0 33.0 ± 4.1	34.0 ± 6.2 32.7 ± 4.0
Percent body fat (%)	TV Stepping Walking	42.4 ± 6.9 41.3 ± 6.3	42.8 ± 6.5^a^ 41.3 ± 6.7^a^	41.8 ± 7.2 40.4 ± 6.8
Waist Circum. (cm)	TV Stepping Walking	105.4 ± 13.6 102.3 ± 8.1	103.4 ± 12.3 101.2 ± 8.3	102.9 ± 12.4^b^ 100.7 ± 8.0^b^
Hip Circum. (cm)	TV Stepping Walking	121.6 ± 12.3 118.9 ± 9.7	119.8 ± 12.4* 118.0 ± 9.6*	119.7 ± 12.5* 117.7 ± 9.6*

* 3 and 6 mo significantly different from baseline; ^a^ 6 mo significantly different from 3 mo; ^b^ 6 mo significantly different from baseline (P < 0.05) Circum. = circumference, PA = physical activity. There were no significant group differences.

Based on self-reported dietary intake, total energy intake was significantly reduced at 3-mo and 6-mo compared to baseline in both groups (P < 0.05), but there was no change in total percent energy from fat. TV-related energy intake and TV-related percent energy from fat decreased over time in both groups (P < 0.05). There was no significant weight loss, or change in BMI in either group over the 6 mo of treatment. Within each group there were significant reductions in body fat percentage, waist circumference, and hip circumference over time (P < 0.05), but no differences between groups (Table 2).

The acceptability of the intervention was high. Attendance at behavioral-based sessions, and phone calls and receipt of newsletters was equivalent between groups across the 6 mo of treatment. Participants in the TV Commercial Stepping group and the Walking group participated in 86.8 ± 29.2% and 87.8 ± 25.6% of the sessions, respectively. During the intervention, finding the time to be physically active, and staying motivated were the two biggest challenges reported by participants in both groups. The Walking group participants also frequently cited weather and lack of a good place to walk as a significant barrier to engaging in PA. Participants in the TV Commercial Stepping group reported surprise at how many steps they could accumulate during commercials. At the end of the 6-mo intervention, 94% of the TV Commercial Stepping participants, and 95% of the Walking participants reported that they planned to maintain their current PA practices.

## Discussion

There are many ways adults can increase their participation in PA [1]. The goal of this pilot study was to examine feasibility and short-term efficacy of a PA program that targeted changing a traditionally sedentary leisure-time behavior (TV viewing) into an active one. The results suggest that a small behavior change, like stepping in place during TV commercials, was both effective and acceptable to adults. At baseline, no participants were meeting the guidelines for aerobic activity (≥150 min/wk). TV Commercial Stepping and Walking both resulted in about 70% of the previously inactive participants meeting the volume requirements of the 2008 PA guidelines, but only the walking group accumulated their PA in continuous bouts of 10 minutes or more. Despite the different approaches to increasing PA, the volume of exercise for both groups was approximately equal. Stepping in place during commercials for least 90 min/d of TV viewing resulted in an increase in daily steps (~3000) roughly equivalent to that which occurred as a result of instructing participants to walk at least 30 min/d (a traditional PA recommendation). Participants in both groups moved from what is considered “sedentary” (<5000 steps/d) to “somewhat active” (7500–9999 steps/d) categories [25]. This novel strategy of TV Commercial Stepping can incorporate exercise into one’s lifestyle, and it has the additional benefit of breaking up sedentary time. Recent evidence suggests that there are health benefits (e.g., a reduction in cardiovascular risk factors) associated with interrupting prolonged periods of sedentary activity with short bouts of standing and physical activity [15,18], and it is of interest that Healy et al. and Dunstan et al. [18,26] have reported that the associations between health and breaking up sedentary time are independent of total sedentary time and moderate-to-vigorous intensity activity. Alternatively, combining TV Commercial Stepping with a traditional exercise program as a way to “boost” overall activity levels may be of interest. As well as boosting overall activity levels, combining TV Commercial Stepping with a traditional exercise program would have the benefit of addressing both prolonged sedentary periods and activity. As these both have independent associations with health this may be a particularly effective ‘two pronged’ approach.

The high rate of attendance to behavioral-based sessions, and high percentage of participants reporting that they planned to continue their current PA practices reflects the high acceptability for both PA interventions. The TV Commercial Stepping intervention was equally acceptable as the Walking intervention. Time and motivation to be physically active continue to be barriers that prevent people from PA compliance. Compliance can be enhanced by home-based exercise programs, and using short bouts rather than long bouts of activity for individuals who “can’t find the time to exercise” [27-30]. TV Commercial Stepping requires no additional time (if worked into the TV programming already being watched), it involves minimal equipment, and can be performed in the comfort of an individual’s home. As an alternative, participants could be encouraged to be physically active (i.e., walk on the treadmill, use the elliptical trainer, stepping in place) throughout the entire 30-min program. However, there was no attempt to test that approach in the current investigation.

An unanticipated outcome of this study was the reduction in TV viewing seen in both groups (Figure 2, and Table 2). Significant evidence demonstrates that TV viewing and PA are inversely related [31-33]. It is possible that TV viewing time was reduced in the Walking group because it was replaced by the 30-min walks, and it may have been reduced in the TV Commercial Stepping group because they associated it with PA. Other possibilities are that the self-monitoring, and subsequent awareness of TV viewing impacted their habits, or there could have been seasonal variation. While it is hypothesized that TV viewing and PA are substitute behaviors, eating and TV viewing appear to be complementary behaviors [34]. An interesting outcome of this study is that both groups reported a reduction of TV-related energy intake and total daily energy intake. However, with only 3 days of dietary data at baseline, 3, and 6 mo, it cannot be determined how participants ate throughout the entire 6-mo period. Also, because dietary intake was self-reported, participants may have under reported energy intake.

Participants maintained their body weight over 6 months, although they had small decreases in adiposity (body fat percentage and circumference measures). These small anthropometric changes are consistent with other short-term (<12 months) exercise-only programs that have resulted in minimal or no changes in weight [35]. The 2008 Physical Activity Guidelines recommend ≥ 150 min/week for overall health, but 150–300 min/week for weight loss [1]. Thus, it is not surprising that the present study, in which participants performed about 150 min per week of moderate PA, resulted in small decreases in percent body fat, but no loss in body weight. Even in the absence of weight loss, increasing PA can result in positive health outcomes [12,36,37]. For this reason, interventions are beneficial if daily step counts increase, independent of whether or not participants achieve weight loss [12,13].

There are several limitations of the present study. This pilot study is limited by its small sample size, disproportionate female gender participation, and limited ethnic diversity. To better generalize the findings of this study, future work will focus on the recruitment of minorities and men. The approach of using TV Commercial Stepping to accumulate PA does have weaknesses; it requires the individual to watch TV, a sedentary behavior. On days when participants did not watch any TV, they were unable to meet their PA goals through this strategy. In the current study design, participants were followed for 6 months, with no follow up after that time point. While we learned that it is possible to intervene during an adult’s TV time, it would have been useful to include a self-report measure of domain-specific sedentary behaviors to systematically assess a wide range of sedentary behaviors. Lastly, there is a need to establish the sustainability of TV Commercial Stepping. Many of the strengths of this innovative study have been highlighted throughout the discussion. The combination of self-report and objectively measured PA is a noteworthy strength. At slower walking speeds some pedometers have been shown to be less accurate. If a participant was stepping in place, walking around the house, or walking outside at a slow walking speed there is the potential that step counts were underestimated. To prevent this from being a major concern, all participants were instructed to “briskly” step in place, or “briskly” walk continuously for the duration their activity bouts. Also, during the first 3 monthly treatment meetings participants demonstrated proper stepping/walking pace. The Omron HJ-303 pedometer used in this study has been shown to provide a valid estimation of steps for walking speeds ranging from 2–8 mph [38]. This study addresses a gap in the literature by testing a potential new strategy in the effort to increase the PA levels and break up sedentary time in the population. It is now evident that stepping during each commercial break is achievable, and it may be worth exploring whether participants might prefer at times to walk in place for the length of a 30-minute program instead of more frequently over the course of a couple of hours. At this early stage of intervention development, the observed outcomes highlight many intriguing issues worthy of future exploration. Without a control group, that received no intervention, it was not possible to determine if either treatment prevented weight gain.

## Conclusions

In conclusion, this is the first study to examine the effects of TV Commercial Stepping as a PA intervention. The results showed that participants in both the TV Commercial Stepping and Walking groups had favorable changes in daily steps, TV viewing, diet, and anthropometrics. Importantly, this study highlights that PA can be performed while viewing TV commercials and this may be a feasible alternative to traditional approaches for increasing daily steps in overweight and obese adults. TV Commercial Stepping should be considered when developing behavioral intervention strategies for increasing exercise participation and breaking up sedentary time in inactive overweight adults. Further research is needed to test the long-term efficacy of the program, its potential impact on PA, dietary choices, and health outcomes.

## Competing interests

Drs.’ Jeremy A. Steeves, David R. Bassett Jr., Eugene C. Fitzhugh, Hollie A. Raynor, and Dixie L. Thompson declare that they have no competing interests.

## Authors’ contributions

JAS was involved in the conception and design of the study, securing funding, implementing and supervising the trial, analyzing data, and drafting the manuscript. DRB was involved in the conception and design of the study, contributing to analyses, in revising the manuscript for important intellectual content, and has given final approval of the version to be published. ECF was involved in the design of the study, contributing to analyses, and revising the manuscript for important intellectual content and has given final approval of the version to be published HAR was involved in the design of the study, contributing to analyses, in revising the manuscript for important intellectual content, and has given final approval of the version to be published. DLT was involved in the conception and design of the study, supervising data analyses, in revising the manuscript for important intellectual content, and has given final approval of the version to be published. All authors read and approved the final manuscript.
